# Efficacy of Phytocannabinoids in Epilepsy Treatment: Novel Approaches and Recent Advances

**DOI:** 10.3390/ijerph18083993

**Published:** 2021-04-10

**Authors:** Aaron M. Farrelly, Styliani Vlachou, Konstantinos Grintzalis

**Affiliations:** 1 Neuropsychopharmacology Division, Behavioural Neuroscience Laboratory, School of Psychology, Faculty of Science and Health, Dublin City University, Glasnevin, Dublin 9, Ireland; aaron.farrelly@dcu.ie; 2 School of Biotechnology, Faculty of Science and Health, Dublin City University, Glasnevin, Dublin 9, Ireland; konstantinos.gkrintzalis@dcu.ie

**Keywords:** epilepsy, treatment, phytocannabinoids, adolescents, young adults, cannabidiol (CBD), delta-9-tetrahydrocannabivarin (Δ^9^-THCV), cannabidavarin (CBDV), cannabigerol (CBG), clinical studies, animal studies

## Abstract

Epilepsy is a neurological disorder mainly characterised by recurrent seizures that affect the entire population diagnosed with the condition. Currently, there is no cure for the disease and a significant proportion of patients have been deemed to have treatment-resistant epilepsy (TRE). A patient is deemed to have TRE if two or more antiepileptic drugs (AEDs) fail to bring about seizure remission. This inefficacy of traditional AEDs, coupled with their undesirable side effect profile, has led to researchers considering alternative forms of treatment. Phytocannabinoids have long served as therapeutics with delta-9-THC (Δ^9^-THC) receiving extensive focus to determine its therapeutic potential. This focus on Δ^9^-THC has been to the detriment of analysing the plethora of other phytocannabinoids found in the cannabis plant. The overall aim of this review is to explore other novel phytocannabinoids and their place in epilepsy treatment. The current review intends to achieve this aim via an exploration of the molecular targets underlying the anticonvulsant capabilities of cannabidiol (CBD), cannabidavarin (CBDV), delta-9-tetrahydrocannabivarin (Δ^9^-THCV) and cannabigerol (CBG). Further, this review will provide an exploration of current pre-clinical and clinical data as it relates to the aforementioned phytocannabinoids and the treatment of epilepsy symptoms. With specific reference to epilepsy in young adult and adolescent populations, the exploration of CBD, CBDV, Δ^9^-THCV and CBG in both preclinical and clinical environments can guide future research and aid in the further understanding of the role of phytocannabinoids in epilepsy treatment. Currently, much more research is warranted in this area to be conclusive.

## 1. Introduction

Epilepsy affects 65 million people worldwide [1]. It is one of the most common neurological disorders, characterised by recurrent seizures [2], which affect individuals of every age [3]. Currently, an array of known causes exists for epilepsy [1] and there have been major advances in our understanding of the disorder, specifically through the lens of biology [4,5]. This enhanced understanding of the underlying physiological mechanisms leading to epilepsy has led to better categorisation of the disorder [3]. Fisher [4] categorised epileptic seizures into focal seizures (limited to one brain hemisphere), generalised seizures (occurring bilaterally over more than one region) and seizures of unknown onset (which cannot be classified as either focal or generalised). Categorizations such as these have led to the refined prescription of antiepileptic drugs (AEDs). These classifications essentially allow medical professionals to prescribe an AED of optimum efficacy based on each patient’s specific form of epilepsy and their age.

The overarching goal of AED prescription is to promote the quality of life via mitigating seizure activity and minimising drug toxicity, with this prescription of AEDs to most epilepsy patients resulting in a significant reduction of epilepsy-related symptoms [6,7]. Currently, there is no cure for epilepsy, nor are there treatments which can prevent the development of the disease [8]. Only therapies exist which treat symptoms of epilepsy, such as seizures. While AED prescription is beneficial for the majority and brings about symptom management, a significant portion of the world’s population presently suffers from the negative effects of epilepsy with no therapeutic drug providing any symptom remission [9]. Many AEDs have been successfully introduced to clinical practice to treat the varying forms of epilepsy, however none of these have substantially aided a significant number of patients. The array of available AEDs only provide seizure control to a segment of the 65 million individuals having epilepsy worldwide [10,11]. AEDs do provide significant benefits to patients when they work, however, there is a major demand to address the problem of ineffective AEDs. This is particularly salient in younger epilepsy patients who currently have no effective treatments available.

Epilepsy in these younger populations arises due to a myriad of factors, the investigation of which can lead to improvements in therapeutic efficacy, which ultimately aids the treatment of the disorder [12]. A cause for concern across the array of epilepsy classifications in children, adolescents and young adults, is pharmacoresistance [13]. Whether epilepsy is of infantile onset, childhood onset or adolescent onset, being resistant to commonly utilised AEDs is a major impediment to current treatment options. In a recent review, numerous derivations of epilepsy (see [12] for more details) all pose significant and nuanced developmental risks to younger populations. Whilst all of the aforementioned forms of epilepsy are unique in their clinical presentation, many share the common characteristic of being persistently resistant to AEDs [12]. Approximately 25% of young epilepsy patients experience this resistance to commonly utilised AEDs [14,15].

Epilepsy deemed as pharmacoresistant is also commonly referred to, as ‘treatment-resistant epilepsy’ (TRE), which is defined as “the failure of adequate trials of two (or more) tolerated, appropriately chosen, and appropriately used antiepileptic drug regimens to achieve freedom from seizures” [16]. The rationale for deeming a patient treatment-resistant after two failed AED trials is based on the likelihood of success with subsequent AEDs, which is low [17,18]. Due to TRE, multidrug combinations and high doses have been adopted in an attempt to compensate for the lack of efficacy of some AEDs in specific populations [1]. These multidrug combinations with high dosages mean that the side effects experienced because of regularly taking AEDs are increased. These side effects include, but are not limited to, double vision, fatigue, difficulty concentrating, memory problems, irritability, depression, extreme body weight fluctuations, reproductive disorders, congenital malformations in pregnancy and an array of behavioural difficulties [19,20,21]. It has been demonstrated that, in younger populations, neurodevelopmental comorbidities resulting from the use of AEDs can bring about more negative effects than the seizures themselves [22,23,24]. AEDs are themselves initially licenced for adult populations, thus further limiting the available data about their safety in younger underage populations [25]. There are multiple limitations inherent in treating epilepsy through these traditional forms. While polytherapy is a response to ineffective treatment, it substantially increases the risk of neurodevelopmental comorbidities [26,27], with the effects present in adolescents and young adults [28,29].

Current research postulates that a ‘new-era’ of treatment for TRE is imminent [30]. Regardless of any imminent ‘new-era’, there is a necessity to continue looking for alternative forms of treatment for the minority of younger individuals suffering from epilepsy whom traditional treatment options have failed. Any novel therapeutic, which can treat epilepsy symptoms in a presently untreatable cohort, whilst also presenting a favourable side-effect profile, should at the very least warrant analysis. Numerous alternate therapies have demonstrated efficacy in their ability to treat epilepsy symptoms [3]. One such potential class of therapeutics is the cannabinoid compounds, which simultaneously demonstrate therapeutic efficacy, favourable side-effect profiles and the ability to counteract previous neurodegenerative damage via inherent neurogenic mechanisms. Across millennia, cannabinoids have been utilised for their medicinal qualities without any knowledge of their underlying molecular pathways [31]. Presently, a great deal more is known about cannabinoids, initiated with the identification of cannabidiol (CBD) and delta-9-tetrahydrocannabinol (Δ^9^-THC) in the 1960s [32,33]. This discovery was subsequently complemented by the discovery of the on-board endocannabinoid system (ECS) in humans, which allowed for the subsequent development of insight surrounding depolarisation-induced suppression of inhibition and excitation (DSI/DSE) mechanisms. This aforementioned process (presented in detail in Section 2 of this review) mediates the effects of plant-derived cannabinoids (phytocannabinoids) *via* endogenous endocannabinoids such as anandamide (AEA) and 2-arichidonyl-glycerol (2-AG) [34,35,36]. It is these modulatory processes that characterise phytocannabinoid activity, giving them the potential to address the current limitations of traditional epilepsy treatment.

**Figure 1:**
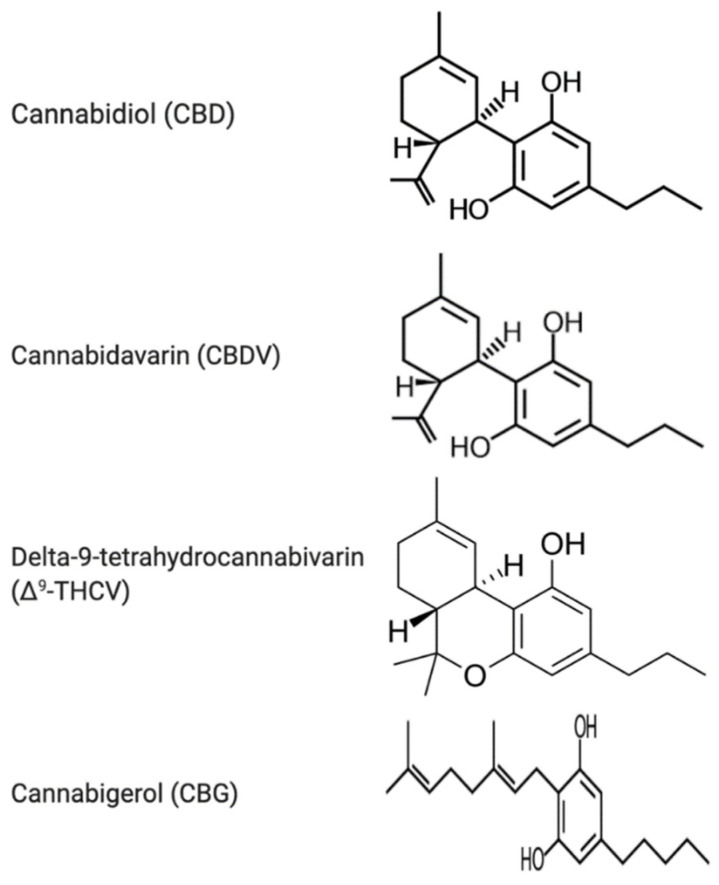
Chemical structures of the phytocannabinoids cannabidiol (CBD), cannabidavarin (CBDV), delta-9-tetrahydrocannabivarin (Δ^9^-THCV) and cannabigerol (CBG).

Most research on the therapeutic effects of phytocannabinoids was traditionally focused on Δ^9^-THC. Recently, however, non-psychoactive cannabinoid derivatives have begun receiving more attention for their therapeutic effects- specifically within the area of TRE. For example, in a randomised, double-blinded, placebo controlled trial on patients with Dravet syndrome (DS) CBD was effective in its ability to reduce seizure frequency (5.9–12.4 per month, *p* < 0.01) [37]. CBD was also shown to significantly reduce seizures specific to Lennox-Gastaut syndrome (LGS) [38,39]. Phytocannabinoid compounds act as potential anticonvulsants, with a range of these derivatives demonstrating anticonvulsant properties [40,41]. This anticonvulsant activity of CBD has not been extended to include other phytocannabinoid derivatives as yet, but a shift in focus is currently moving towards the therapeutic potential of other underexplored phytocannabinoids [42]. The overall aim of this review is to explore current advances in this direction with respect to the treatment of epilepsy. Specifically, the current review intends to achieve this *via* an exploration of the molecular targets underlying the anticonvulsant capabilities of novel phytocannabinoids (See Figure 1). Further, the review will also provide an exploration of current pre-clinical and clinical data as it relates to specific phytocannabinoids and the treatment of epilepsy symptoms.

These non-psychoactive phytocannabinoids, cannabidiol (CBD), cannabidavarin (CBDV), delta-9-THCV (Δ^9^-THCV) and cannabigerol (CBG), all warrant analysis through the lens of epilepsy due to their anticonvulsant characteristics combined with favourable side-effect profiles [42]. Furthermore, hypotheses that phytocannabinoids also present a solution to the neurodegenerative deficits associated with epilepsy are realistic. A clear neuroprotective/neurogenerative characteristic has been identified and associated with these phytocannabinoid compounds [43], with accumulating evidence considering phytocannabinoids as neuroprotective [44]. This review will also elucidate these neurogenic mechanisms and their implications for epilepsy treatment in younger populations.

## 2. The Endocannabinoid System and Its Modulation by Phytocannabinoids

The ECS is made up of three main components including receptors, ligands and enzymes. The traditional ECS receptors are known as CB_1_ and CB_2_. These receptors belong to the G-protein-coupled receptor (GPCR) proteins [34]. CB_1_ receptors are primarily expressed in the central nervous system (CNS), while research demonstrates that CB_2_ receptors are more expressed throughout the immune system [45]. Research has also shown CB_2_ receptor expression to occur in the CNS [46,47,48]. Except for CB_1_ and CB_2_ receptors, numerous other receptor channels have been implicated in ECS signalling. Remaining within the GPCR realm, it has been demonstrated that GPR55 and GPR18 are specifically implicated in phytocannabinoid modulation [49]. Transient receptor potential channels (TRP), which are a class of membrane proteins involved in an array of signal transduction pathways, have also been implicated in ECS modulation. Specifically, transient receptor potential vanilloid (TRPV), transient receptor potential ankyrin (TRPA) and transient receptor potential melastatin (TRPM) subfamilies have all been implicated [50,51,52]. Each of these channels contains further, more specific receptors with demonstrated activity relating to the ECS. Research has also demonstrated that broader ECS communication can be mediated by peroxisome-proliferator-activated receptors (PPARs) [53,54]). These nuclear hormone receptors control the transcription of specific genes. The activation of specific PPAR family receptors (PPARα and PPARγ) has been associated with anti-inflammatory and neuroprotective characteristics of exogenous phytocannabinoids [49]. The central consideration in this instance is that the ECS has a wide breadth in terms of cellular receptors, which bring about effects. Ultimately, these receptors all play a key role in the modulation of neural activity.

**Figure 2:**
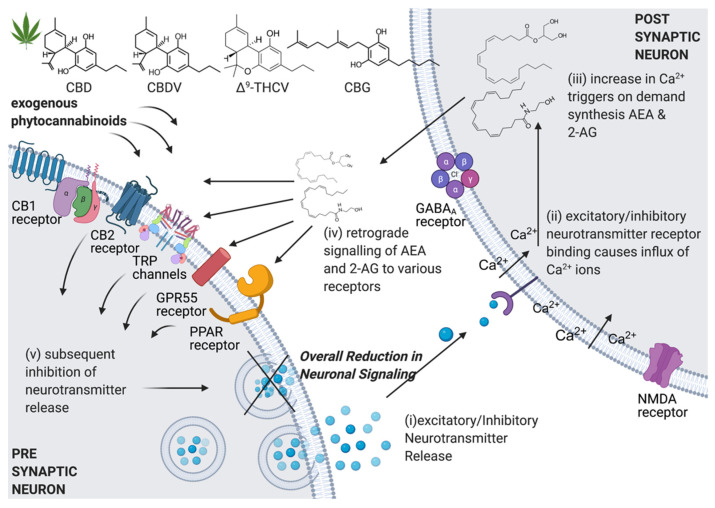
Synaptic transmission of the ECS: (**i**) Excitatory/inhibitory neurotransmitter release into the synapse (**ii**) Neurotransmitter binding to post-synaptic neuron (**iii**) Depolarisation causes an influx of calcium ions that initiates on-demand synthesis and release of endocannabinoids (**iv**) Binding of endocannabinoids to various pre-synaptic receptors leads to subsequent modulations in neurotransmitter release (**v**) Inhibition of further synaptic communication reduces seizure activity downstream.

Another component of the endogenous ECS, which is critical for the modulation of neural transmission is the ECS-specific ligands that bind to receptor channels. AEA and 2-AG are the two primary ECS ligands and they act as retrograde neurotransmitters. This essentially means that depolarisation of the post-synaptic terminal triggers their release, upon which they travel back (i.e., in a retrograde manner) from the post synaptic terminal to the pre-synaptic terminal. On the pre-synaptic terminal they bind primarily to CB_1_ receptors and the TRP superfamily of ion channels, which mediate the transduction of a plethora of intracellular stimuli (see Figure 2). The ligands are regulated by the enzymes fatty acid amide hydrolase (FAAH) and monoacylglycerol lipase (MAGL) [55]. What makes these two enzymes unique is that they are synthesised in an on demand fashion in post-synaptic neurons. The enzymes are produced from membrane phospholipid precursors in the post-synaptic terminal [56]. The synthesis of these enzymes from phospholipid precursors depends on intracellular increases in Ca^2+^ levels, which occur because of previous neurotransmission [57]. Ultimately, the ECS receptors, ligands and regulatory enzymes communicate to regulate neural activity. This communication results in retrograde signalling known as DSI. DSI occurs when the post-synaptic terminal depolarises, causing a subsequent reduction of GABA-mediated inhibitory transmission. This is characterised as a short-term synaptic depression [58]. Similar to DSI, DSE has also been characterised. In DSE, rather than the inhibition of GABA transmission, inhibition of glutamate-mediated transmission occurs which subsequently inhibits excitatory potentials [59].

This retrograde signalling characterises the ECS as a system that operates *via* a mechanism, which can treat disorders characterised by aberrant neural activity. Through the manipulation of DSI and DSE in different brain regions there is a potential to alter the activity of the CNS. Seizure activity has been defined as ‘Paroxysmal’—meaning neural networks are experiencing too much excitation coupled with minimal inhibition. This paroxysmal activity can be regulated by the ECS, with its implication in activity-dependent long-term depression of glutamatergic and GABAergic synaptic transmission [60,61]. Due to its modulatory capacity, the ECS has become a systemic target for treating various neurological disorders. Furthermore, the existence of phytocannabinoid compounds that can regulate ECS activity and broader intracellular communication provides further novel targets for treating neurological disorders. With the ECS characterised by an ability to alter neural communication, the administration of phytocannabinoids, which regulate this ECS may provide therapeutic benefits in epilepsy. CBD has demonstrated an ability to modulate ECS-related machinery in a manner accommodating seizure reduction in humans with severe forms of TRE [15,37,38,62]. With this efficacy inherent in the phytocannabinoid CBD demonstrated, the exploration of other phytocannabinoids for their potential anticonvulsant capability is warranted, specifically CBDV, Δ^9^-THCV and CBG. By focusing on their specific molecular targets, and both preclinical and clinical observations, this review intends to highlight which phytocannabinoids warrant further investigation from the perspective of treating epilepsy in young populations.

## 3. Anticonvulsant Effects of Phytocannabinoids

To understand epilepsy and determine whether specific exogenous compounds provide anticonvulsant efficacy, numerous models have been created to simulate epilepsy in animals. These models were created to provide further comprehension of the complex mechanisms underlying seizure activity and epileptogenesis which cannot be solely understood through research in humans. For a comprehensive overview of animal models used in epilepsy research there are numerous salient sources (for examples, see [63,64,65,66]). These specific animal models are evaluated in terms of their epilepsy simulating ability [67], which allows the identification of appropriate models for specific forms of the disease [68].

Models primarily utilised by research contained in this review include the pentylenetetrazol (PTZ) model, the maximal electroshock (MES) model and the audiogenic seizures (AS) model, as well as the pilocarpine model. Various other models of epilepsy exist in animals, however, within the studies on phytocannabinoids, these models appear to be most commonly utilised. The following is a presentation of four specific phytocannabinoids and their individual characteristics, which make them novel targets for treating epilepsy. First, an analysis of the molecular targets which are said to underpin the compounds’ anticonvulsant capability will occur. A presentation of evidence demonstrating this anticonvulsant activity will then follow.

### 3.1. Cannabidiol

CBD is the most commonly analysed phytocannabinoid after its psychotropic counterpart Δ^9^-THC [69]. The CBD-based therapeutic Epidoliex^®^ (Greenwich Biosciences, Carlsbad, CA, USA) has demonstrated significant merit in addressing TRE. Based off these findings, CBD was given approval by the Food and Drug Administration (FDA) in the USA for treating epilepsy in 2018.

#### 3.1.1. Molecular Targets of CBD

More than 65 molecular targets have been reported in the literature for CBD. A relatively small number of these targets represent a plausible explanatory mechanism for the compound’s anticonvulsant activity [70].

At low micromolar concentrations CBD blocks the equilibrative nucleoside transporter (ENT), the orphan G-protein-coupled receptor (GPCR55), and the transient receptor potential of melastatin type 8 (TRPM_8_) channel [40]. CBD also enhances the activity of serotonin receptors (specifically the 5-HT_1a_), the transient receptor potential of Ankyrin type 1 channel (TRPA_1_), and second messenger cascades *via* its modulatory effect on intracellular calcium levels [71]. At higher micromolar concentrations CBD has been demonstrated to activate the nuclear peroxisome proliferator-activated receptor-v (PPARV) and the transient receptor potential of vanilloid type 1 (TRPV_1_) and type 2 (TRPV_2_) channels [71]. Its modulation of tumour necrosis factor alpha (TNF-alpha) [72] and its inhibition of adenosine reuptake [73] also provide viable explanations as to its efficacy in treating epilepsy.

The activity of CBD at GABA_A_ receptors is also an interesting target for this compound. GABA mediates fast inhibitory neurotransmission in the central nervous system *via* its action on GABA_A_ receptors. These receptors have been previously implicated in various neurological and psychiatric disorders including epilepsy [74]. Recently, CBD has been postulated to exert its therapeutic effects through GABA_A_ receptors, as disinhibition of GABAergic neurons provides therapeutic alterations in downstream neural activity. Opposite to these disinhibitory effects at GABA_A_ receptors, CBD also inhibits N-methyl-D-aspartate (NMDA) receptors [75]. NMDA receptors have been implicated in memory processes [76], but their inhibition has also been linked to other neurological disorders such as epilepsy. They present another novel target by which CBD may enact its anticonvulsant capability. Further, CBD also inhibits the cellular uptake of fatty acid amide hydrolase (FAAH) and the degradation of AEA. CBD also potentiates the beneficial effects of Δ^9^-THC whilst simultaneously reducing its psychoactivity. This phenomenon has been coined the ‘entourage effect’ and is not limited to the interaction of CBD with Δ^9^-THC [77]. Research has also implicated the importance of large conductance calcium-activated potassium (BK) channels in the activity of CBD. It has been demonstrated that CBD acts as an effective agonist to BK channels bringing about significant anticonvulsant effects [78].

Whilst these previously mentioned pathways have been elucidated to illustrate the effects of CBD, the exact means by which CBD exerts its anticonvulsant capacity remains unknown [40]. It is clear however that CBD is a pleiotropic compound and its efficacy in epilepsy stems from these multifaceted effects. It has also been hypothesised that CBD reduces neuronal excitability and transmission during seizure through the modulation of intracellular calcium, which acts as the second messenger in signal cascades. While being essential for the synthesis of the ECS ligands AEA and 2-AG, this modulation of calcium levels also promotes potential therapeutic interactions with targets such as TRP channels [79], GPCR55 channels and voltage dependent anion-selective channels (VDAC_1_) [80]. Such interactions may further explain the anticonvulsant effects of CBD.

Alternatively, the therapeutic effects of CBD could be explained by its polyphenolic nature [40]. The repeating polyphenolic moieties of CBD make it a powerful antioxidant and imbue the compound with anti-inflammatory characteristics. These neuroprotective and generative effects of CBD further illustrate the multifaceted nature of the compound and its ability to promote various therapeutic effects, which can aid the symptoms of epilepsy. Overall, much remains to be understood about the specific mechanisms involved in the effects of CBD. Further analysis of this compound is essential, with a specific focus on identifying epilepsy-related brain regions and how CBD works within them.

#### 3.1.2. Pre-Clinical Evidence of CBD Efficacy

The anticonvulsant effects of CBD have been demonstrated across numerous animal models of epilepsy in recent decades. In rats for example, across both MES and AS models, CBD anticonvulsant capability has been demonstrated at varying doses (12 and 17 mg/kg) [81] (Table 1). As well as sole efficacy, CBD was also compared with other AEDs in separate experiments still utilising the MES model. It was observed that CBD was as potent as the AED phenytoin and had slightly less efficacy compared to another AED, phenobarbital. CBD in this instance was also found to be more effective than both AEDs trimethadione and ethosuximide [81].

The efficacy of CBD was also demonstrated in the PTZ model of acute epilepsy when 60 mg/kg of the compound was administered intraperitoneally (IP). This administration of CBD subsequently reduced the tonic seizures induced by PTZ [82]. In this instance the researchers explained CBD efficacy through its ability to disinhibit GABA_A_ receptors. The disinhibitory effect at GABA_A_ receptors has been replicated and discussed in more contemporary research [83]. This consistency in preclinical findings across time lends further validity to the therapeutic efficacy of CBD in epilepsy. Results obtained across animal models of the disease further bolster this validity.

Researchers further observed the efficacy of CBD within the PTZ model of seizure. CBD acted as an effective agonist to voltage-gated potassium channels. These channels are responsible for the conductance of large quantities of potassium ions across the cell. The researchers in this instance concluded that such activity promoted significant anticonvulsant effects in rodents [78]. Continued analysis of CBD activity in mice without these voltage-gated potassium channels [78] pointed further to their potential in bringing about CBD anticonvulsant properties. These rodents in which the voltage-gated potassium channel was knocked-out provide preliminary evidence for the role these channels play in the anticonvulsant activity of CBD.

Preclinical research has also attempted a more chronic elucidation of CBD’s efficacy as an anticonvulsant. Using the PTZ model of epilepsy, it was found that a reduction in seizure activity could be garnered by the administration of varying doses (20 and 50 mg/kg) of CBD over a 28-day period. In tandem with this lower seizure activity, CBD-treated rodents also had lower neuronal death in CA_1_ and CA_3_ hippocampal regions [84]. This shows evidence of the postulated neuroprotective characteristics of phytocannabinoids. CBD was also observed in this instance to have downregulating effects on NMDA subunit 1 expression in the hippocampus, further showing the multifaceted targets of the phytocannabinoid [84]. Preclinical evidence of CBD’s anticonvulsant capability across animal models of epilepsy coupled with comprehensive data about its safety [85] ultimately allowed clinical trials to start in human participants. These clinical trials eventually lead to the therapeutic efficacy of CBD being realised across human populations including adolescents and young-adults [86,87,88].

#### 3.1.3. Clinical Evidence of CBD Efficacy

CBD was first tested in human populations with epilepsy in the late 1970s [96]. Mechoulam and Carlini [89] conducted initial placebo-controlled research with a small sample (*n* = 9) who presented with medically uncontrolled epilepsy (Table 1). Participants were randomised to receive 200 mg/day of CBD for 3 months in tandem with their usual battery of AEDs. In the CBD-treated group (*n* = 4), two patients achieved a full recovery from epilepsy symptoms with the third patient demonstrating a partial recovery. The fourth patient in the epilepsy group was unresponsive to treatment. The remaining participants in the placebo group (*n* = 5) saw no significant improvements in epilepsy-related symptoms. A key finding of this initial trial as well as efficacy, was the fact that no patients in the CBD group experienced any side effects [89]. With drastic side effects of AEDs posing significant problems for epilepsy treatment, demonstration of minimal side effects by CBD was positive. In another clinical trial, Carlini and Cunha [90] reported improvements in patients with temporal lobe epilepsy through the administration of 200 or 300-mg CBD daily for 4 months. Again, as previously, side effects were minimal with only somnolence being reported. With this early replication of therapeutic efficacy in tandem with a lack of side effects, it becomes clear why CBD became the primary phytocannabinoid of focus for many researchers.

More recently the efficacy of CBD has been further replicated in human populations including adolescents and young-adults with severe childhood-onset epilepsy [15]. In this open-label trial conducted over 12 weeks, doses of 25 mg/kg or 50 mg/kg of CBD were administered to patients (Aged 1–30) in tandem with their prescribed AED regiment. The CBD-treated groups showed a reduction in seizures. Utilising number of ‘drop seizures per month as the outcome variable, a 36.5% reduction in these seizures was seen in the CBD-treated groups. Similar to other trials in human populations, the side effects experienced were also much less pronounced in the CBD-treated groups compared with groups receiving traditional AEDs alone. Interestingly, these favourable side effect profiles associated with CBD administration in tandem with AEDs were more common in the groups who were using the traditional AED clobazam [15].

Further trials on the efficacy of CBD have also been conducted specifically in children and younger adult populations. In another trial, a sample consisting of children and young adults who presented with Dravet Syndrome (DS) received 20 mg/kg of CBD (or placebo) in tandem with their standard AED drug regimen for 14 weeks. The sample showed a significant reduction in seizures, with 5% of this sample becoming seizure free after the 14-week period [37]. In another trial of similar nature, Lennox Gastaut Syndrome (LGS) was the common denominator of a sample (age 2–55 years), which contained infants, adolescents, young adults, adults and older adult patients. These participants received varying doses (10 or 20 mg/kg) of CBD in tandem with their regular regimen of AEDs for 14 weeks. Drop seizures were implemented as the outcome measure. A 41.9% reduction in drop seizures was recorded in the 20 mg/kg CBD-treated group, and a 37.2% reduction in drop seizures was recorded in the 10 mg/kg CBD-treated group. The placebo group had a 17.2% reduction in drop seizures [38].

Overall, the evidence for CBD’s efficacy in treating epilepsy has been shown and replicated across various forms of TRE. Of paramount importance are the clear benefits this phytocannabinoid has brought to younger people suffering from TRE. Since its approval as an alternate AED [97], CBD has also shown favourable side-effect profiles across different populations including adolescent and younger adult samples [98]. This demonstration of CBDs ability to treat previously resistant forms of epilepsy should prompt researchers to begin analysing the potential merit of other structurally similar phytocannabinoids.

### 3.2. Cannabidivarin (CBDV)

Cannabidivarin (CBDV) is another phytocannabinoid and direct CBD analogue derived from cannabigerovarin. CBDV has shown anticonvulsant effects and a favourable pharmacokinetic profile similar to CBD [42,99]. From a molecular perspective CBDV works through various mechanisms, which parallel other novel phytocannabinoids discussed in this review, such as CBD and CBG.

#### 3.2.1. Molecular Targets of CBDV

The mechanisms by which CBDV acts are diverse. CBDV inhibits the reuptake of AEA and 2-AG at micro- and nanomolar concentrations, respectively [91,92]. Research has also shown the strong role of ECS membrane transport mechanisms in overall brain homeostasis, with a specific focus on the role AEA reuptake inhibitors play in regulating this process [100]. The synthesis of explicit ‘probe’ inhibitors such as WOBE437 and RX-055 for inhibiting AEA reuptake shows the significant role that reuptake inhibition plays in regulating brain activity [101]. With CBDV known to act as an enzyme reuptake inhibitor, similar to other phytocannabinoids, it is hypothesised that CBDV acts as an effective anticonvulsant.

Except for the endocannabinoid reuptake inhibition, CBDV also activates TRPV_1_ and TRPV_2_ channels as well as TRPA_1_ channels [91,102]. TRP proteins essentially form cation channels, which, upon activation, depolarise the membrane potential, leading to either activation or inactivation of voltage-gated ion channels and mechanisms that influence Ca^2+^ signalling [103]. These aforementioned processes control diverse cellular functions [104]. With diverse effects at a cellular level, TRP channels and subfamilies are highly expressed and play a critical role in brain communication [105]. It has been shown that this role is essential to processes characteristic of epilepsy. Dysregulation of the previously mentioned channels leads to cortical spiking and wave seizures, which are definitive signs of epilepsy [106]. The fact that CBDV has been shown to dose dependently activate these channels (TRPV_1_, TRPV_2_ and TRPA_1_) makes it a novel target for treating epilepsy symptoms.

#### 3.2.2. Pre-Clinical Evidence of CBDV Efficacy

CBDV efficacy has been investigated across numerous animal models of epilepsy. It has anticonvulsant effects across the MES, AS, PTZ and Pilocarpine models of epilepsy [81,90] (Table 1). Work in the MES model has shown that animals who received 100 or 200 mg/kg demonstrated significantly less hindlimb extension (a key phenotypic marker of seizure severity in animals) after seizure induction. This result provided initial behavioural observations that demonstrate CBDVs anticonvulsant characteristics. In this same experiment any animal that was vehicle-treated demonstrated insignificant changes in hindlimb extension [81]. The incidence of tonic-clonic seizures was also significantly low in CBDV-treated mice compared to the vehicle-treated group. Overall, these findings within the MES model showed that the percentage of seizure-free rodents was significantly higher after administration of 200 mg/kg of CBDV, with this higher dose bringing about more robust anticonvulsant effects.

Hill and colleagues [91] further showed that CBDV significantly reduced epilepsy-related symptoms. Working within the PTZ model of epilepsy, CBDV was administered at doses of 200 mg/kg, which brought about reductions in overall seizure severity. This same dose (200 mg/kg) also produced significant reductions in rodent mortality. In this PTZ model of epilepsy, results showed that 33.3% of rodents in the 200 mg/kg group displayed no sign of seizure. This contrasted with only 6.7% of rodents in the vehicle group displaying no signs of seizure. The seizure onset was shown to be significantly delayed in CBDV-treated mice. In both of these previously discussed analyses, CBDV was the sole compound being administered, thus, the results are more reflective of CBDVs pure efficacy. While CBDV’s direct mechanisms of action remain elusive, its significant ability to treat the symptoms of chemically-induced convulsions in this instance can be more readily attributed directly to CBDV’s actions as it was the sole compound administered.

Hill and colleagues [91] conducted further experiments where CBDV was analysed experimentally in tandem with participants’ current AED regimens. This directly contrasts the researchers’ previous analyses where CBDV was the sole compound administered. CBDV had anticonvulsant effects and was well tolerated when co-administered with AEDs.

Hill and colleagues [92] further analysed the anticonvulsant properties of a cannabis-derived botanical drug substance rich in CBDV across various animal models of epilepsy. At doses of 200 mg/kg and 275 mg/kg, seizure severity was suppressed, with the larger dose significantly reducing rodent mortality. This modified compound rich in CBDV was shown to have strong anticonvulsant effects across the PTZ and AS models. Less efficacy was demonstrated in the pilocarpine model when data were compared with the PTZ and AS results [92]. From these experiments it was shown that CBDV can have positive effects on epilepsy symptoms. The administered compound also contained other phytocannabinoids, such as delta-9-tetrahydrocannabivarin (Δ^9^-THCV). Through containing such a mixture of phytocannabinoids the authors concluded that the compound affinity for CB_1_ receptors was significantly increased, even so it was determined that this modified CBDV compound showed promising results as an anticonvulsant. With results showing the efficacy of CBDV as an anticonvulsant emerging in preclinical research, it became important to begin translating these findings to research on humans.

#### 3.2.3. Clinical Evidence of CBDV Efficacy

CBDV has received minimal attention in human research regarding its ability to treat epilepsy. Human trials have been conducted in other disorders and specific procedures have also determined the pharmacokinetic profile of numerous oral CBDV formulations in humans (25, 75, 200, 400 and 800 mg/day). Overall, it has been demonstrated that CBDV is well tolerated by participants and rapidly metabolised in the liver [107] (Table 1).

Trials have been conducted, which evaluated the characteristics of CBDVs efficacy and safety in human participants. Morano and colleagues [93] conducted a phase 2 trial where a plant-derived purified formulation of CBDV (GWP42006) was administered to adult patients who suffered from specific TRE. In a randomised block design participants received 400-mg CBDV over a treatment period of 14 days. It was observed that CBDV did not significantly reduce seizure instances [93]. However, data indicated further that CBDV was a safe compound that could be used in human participants [108]. Whilst CBDVs efficacy was not demonstrated, the trial design contained weaknesses that merit further investigation. For example, the utilisation of a purified formulation of CBDV casts doubt on the validity of these results to represent CBDVs overall efficacy in epilepsy. The inefficacy of purified cannabinoids has previously been demonstrated with the use of purified CBD in comparison to a CBD-rich formulation [77].

Future research should focus on CBDV-rich formulations whilst also addressing the dearth of research in younger populations. Future trials of CBDV in epilepsy should target specific populations (e.g., adolescent/young adult) to avoid the sampling limitations experienced in the previous trial. Homogenous samples of adolescent and younger adult TRE patients should be considered as these samples are in major need of an effective therapeutic. Different formulations (‘purified’ versus ‘rich’) of CBDV should also be utilised to further capture CBDV’s efficacy, as has been done with CBD [77,93]. A patent describing a CBDV-based compound has been approved [109], however while this is a promising advancement, CBDV still needs to be further tested for epilepsy treatment.

### 3.3. Delta-9-Tetrahydrocannabivarin (Δ^9^-THCV)

**Figure 3:**
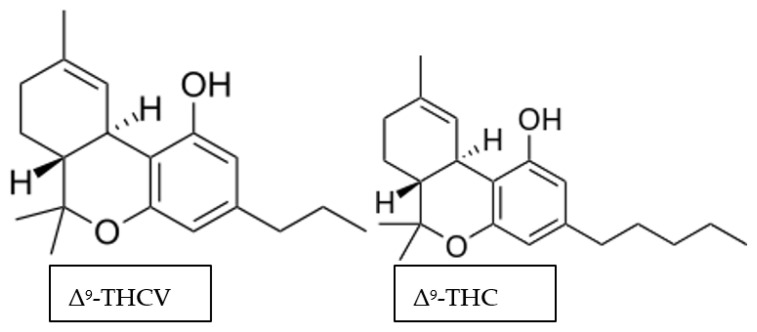
Chemical structure of the non-psychotropic delta-9-tetrahydrocannabidavarin (Δ^9^-THCV) and the psychotropic delta-9-tetrahydrocannabinol (Δ^9^-THC).

Δ^9^-THCV is a phytocannabinoid with structural similarity to the psychotropic phytocannabinoid Δ^9^-THC (Figure 3). Δ^9^-THCV has a shorter side chain on its phenyl-ring, but still has the capability to traverse the blood brain barrier (BBB) [42]. Also, CBDV isomerises into Δ^9^-THCV, making CBDV a biosynthetic precursor of this compound. With research pointing to the efficacy of CBDV in epilepsy, it is prudent to hypothesise that Δ^9^-THCV also holds therapeutic promise for treating epilepsy.

#### 3.3.1. Molecular Targets of Δ^9^-THCV

Δ^9^-THCV has diverse pharmacokinetics and displays tissue-dependent effects [42,71]. Early research on Δ^9^-THCV demonstrated that it was capable of inducing seizure-like activity in rodents [110]. More recent research has begun utilising specific assays ([^35^S]GTP-yS- binding assay) to further determine the molecular underpinnings of Δ^9^-THCVs activity. The [^35^S]GTP-yS- binding assay has aided in uncovering the molecular effects of various specific compounds [111].

Through the utilisation of the [^35^S]GTP-yS- binding assay, it has been demonstrated that Δ^9^-THCV exhibits antagonist actions at both CB_1_ and CB_2_ receptors [112]. More specifically, this ECS receptor action is region specific, with Δ^9^-THCV acting as an antagonist in the cerebellum and piriform cortical membranes [113]. *In vitro* investigations of Δ^9^-THCV’s mechanisms have further confirmed the functional antagonism observed *in vivo* [112]. Except for region-specific effects, dose-dependent responses through varied mechanisms have also been observed with higher concentrations of Δ^9^-THCV inhibiting seizure like activity through a non-CB_1_ receptor-mediated mechanism [112]. Despite preliminary evidence of Δ^9^-THCVs activity via ECS receptors, the specific functional mechanisms by which Δ^9^-THCV exerts its action are still not fully understood [114]. Through ‘patch clamp’ and multi-electrode array analyses in rodent brains, it was concluded that Δ^9^-THCV acts similarly to selective CB_1_ receptor antagonists. Essentially Δ^9^-THCV modulates inhibitory neurotransmission at cellular and network levels [114]. Δ^9^-THCV was observed to increase inhibitory neurotransmission by increasing GABA release [71].

It has been observed that Δ^9^-THCV behaves as both a CB_1_ and CB_2_ orthosteric ligand in binding assays, and as a neutral CB_1_ receptor antagonist in functional assays [115]. In more recent analyses of the compound, McPartland and colleagues [115] characterise Δ^9^-THCV as a high affinity CB_1_ receptor ligand and potent antagonist *in vitro*, which can still achieve antagonistic effects *in vivo*, but not with the same efficiency as observed *in vitro*. To elucidate Δ^9^-THCV’s mechanisms of action in depth and overcome current limitations in understanding, additional *in vivo* research must be conducted on this compound [115].

#### 3.3.2. Pre-Clinical Evidence of Δ^9^-THCV Efficacy

Δ^9^-THCV has been established as a suitable agent for treating various disorders [116,117,118,119] with its efficacy and safety being demonstrated across age groups including adolescent and younger adult populations [119]. Whilst Δ^9^-THCV has been trialled as a potential therapeutic in many disorders, epilepsy is not one of these disorders. Direct analyses that specifically attempt to understand Δ^9^-THCV’s capability as an anticonvulsant are sparse, with most preclinical research data about Δ^9^-THCVs potential as an anticonvulsant stemming from *in vitro* designs. These designs are more concerned with mapping Δ^9^-THCVs mechanisms of action, rather than analysing its explicit efficacy as an anticonvulsant. However, attempts have been made to bridge the gap from *in vitro* to *in vivo*.

In an experiment focusing on both *in vitro* (Piriform-cortex-brain-slice model) and *in vivo* (PTZ Seizure model) methods, Hill and colleagues [94] investigated the anticonvulsant effects of Δ^9^-THCV (Table 1). From an *in vivo* perspective Δ^9^-THCV was administered to rodents either pre-seizure induction, or during seizure activity, at doses of 10 μM and 10–50 μM, respectively. Post seizure, the effects of each of these doses were analysed to generate data on their respective abilities to mitigate the seizure activity. A dose >20 μM of Δ^9^-THCV could significantly reduce seizure activity in rodents. When Hill and colleagues [94] compared these dosages, to the dosage required to mitigate seizure activity *in vitro*, it was clear that less Δ^9^-THCV was necessary in order to bring about anticonvulsant effects *in vitro. In vitro* piriform cortex slices only needed a dose of 10 μM to induce anticonvulsant effects. Overall, Hill and colleagues [94] concluded that 0.25 mg/kg of Δ^9^-THCV significantly reduced seizure incidence in the PTZ model of epilepsy. Results of this experiment point to a preliminary confirmation that Δ^9^-THCV possesses anticonvulsant characteristics. This promising preliminary data received little follow up, resulting in a dearth of available information about Δ^9^-THCVs ability as an anticonvulsant. Whether Δ^9^-THCV is capable, or incapable of acting as a reliable anticonvulsant across models of epilepsy remains to be seen.

In a review article, McPartland and colleagues [116] concluded that Δ^9^-THCV is best suited to treating diabetes, which is warranted as plenty of data exists to support this claim. However, little evidence is currently available to conclude that Δ^9^-THCV is not effective in treating the symptoms of epilepsy.

### 3.4. Cannabigerol (CBG)

CBG is one of the major phytocannabinoids present in *Cannabis sativa*, which was isolated, characterised and synthesised by the same research team who conducted the pioneering work on the main psychoactive constituent (Δ^9^-THC) of *Cannabis sativa* [33]. Assays have demonstrated that unlike its psychotropic counterpart Δ^9^-THC, CBG is non-psychoactive [120]. Novel mechanisms of action, as well as its efficacy in epilepsy, have been considered by researchers.

#### 3.4.1. Molecular Targets of CBG

Analyses have revealed diverse molecular targets of CBG, with some of these targets providing possible explanations for the potential anticonvulsant effects of the compound. In rodents, it has been demonstrated that CBG acts as an agonist for both CB_1_ and CB_2_ receptors [71]. This simultaneous activity of CBG at both primary receptors of the ECS was confirmed by the observation of K_i_ values between 300 and 500 nM at both CB_1_ and CB_2_ receptors. Apart from demonstrating an affinity for CB_1_ and CB_2_ receptors, CBG has also affected various other pathways. De Petrocellis and colleagues [51] observed that CBG acts as an agonist of TRPA_1_, TRPV_1_, and TRPV_2_ channels, whilst also acting as an antagonist of TRPM_8_ channels. This study also demonstrated that CBG can act as an endogenous endocannabinoid reuptake inhibitor, inhibiting the reuptake of anandamide in a manner similar to other phytocannabinoids [51]. CBG also directly opposes the effects of other phytocannabinoids; for example CBG opposes the effects of CBD at the serotonin (5-HT_2A_) receptor through antagonistic action [121]. Furthermore, CBG blocks the activity at 5-HT_1A_ receptors [122]. This proclivity to modulate the activity at serotonin receptors is a key characteristic of CBG activity. Furthermore, CBG activates α_2_-adrenoreceptors, implicating it in the inhibition of noradrenaline uptake [122].

More recently research on CBG reports that effects can be observed through the PPAR pathway, with CBG higher cellular concentrations enhancing the transcriptional activity of PPARγ, which is responsible for the expression of a large number of genes controlling various cellular mechanisms including cell proliferation [123]. Recent investigations have also implicated GPR55 in the activity of CBG. By utilising the [^3^H]-CP-55940 and [^3^H]-WIN-55, 212-2 receptor binding assays, Navarro and colleagues [124] were further able to show CBGs molecular targets. Through the investigation of the binding properties of CBG with a specific focus on CB_1_ and CB_2_ receptors individually, as well as a focus on CB_1_-CB_2_ receptor heteromers and the complexity of communication involved in receptor crosstalk [125], researchers could illustrate particular aspects of CBG activity at ECS related receptors. They showed that CBG modulated signalling mediated by CB_1_ and CB_2_ receptors as well as CB_1_-CB_2_ receptor heteromers. This modulation was even shown to occur at low micromolar concentrations of CBG (0.1–1 μM), subsequently bringing about changes in line with the characteristics of a partial agonist. Navarro and colleagues [124] also illustrated CBG’s activity on deeper signal transduction pathways such as cyclic adenosine monophosphate (cAMP), protein kinase R-like ER kinase (PERK) and β-arrestin. This research group [124] subsequently concluded that the underlying molecular mechanisms about CBG activity remain uncertain.

#### 3.4.2. Preclinical Evidence of CBG Efficacy

Preclinical research has provided pharmacokinetic analyses of CBG to understand its characteristics and activity *in vivo* [42]. Intraperitoneal injection was found to yield considerably higher CBG concentrations in rodent blood plasma and brain tissue compared to oral administration routes. As well as this, CBG was readily observed crossing the BBB, due to its lipophilic nature, with T_max_ values between 30 and 120 min [42].

In terms of anticonvulsant capability, the literature is unable to state whether CBG is an effective anticonvulsant due to sparse analyses. Hill and colleagues [95] assessed the anticonvulsant effects of CBG *in vivo*. Specifically, the effect of CBG was assessed in Wistar rats where seizures were induced and anticonvulsant capability was assessed using the PTZ model of acute epilepsy (Table 1). Doses of 50–200 mg/kg CBG demonstrated no anticonvulsant effects on PTZ-induced seizures, but did demonstrate an effect on Na^+^ channels to a similar degree as CBD. This prompted the research group [95] to conclude that similar to CBD, CBG can block Na^+^ channels at micromolar levels, but cannot provide concomitant mitigation of seizure activity [95].

There is a lack of pre-clinical research specifically analysing CBG’s efficacy as an anticonvulsant, some research has however began identifying CBG’s effects in relation to other phytocannabinoids. For example, CBG has been evaluated for its potential to reverse the effects enacted by CBD. Rock and colleagues [121] demonstrated that CBG opposes the effects of CBD at the serotonin (5-HT_2A_) receptor. With CBD known to regulate various physiological effects via its agonist effect on the 5-HT_2A_ receptor, CBG opposes these effects via antagonistic action at the 5-HT_2A_ receptor, subsequently reducing side effects associated with the administration of specific compounds such as nausea and vomiting. With CBG shown here to potentially work as an antagonist of serotonin receptors, and other data leading to postulations that 5-HT_2A_ receptor signalling is implicated in the control of neuronal excitability (through GABAergic, monoaminergic and glutamatergic neurotransmission), it merits investigating the possible anticonvulsant effects of CBG through its direct modulation of activity at 5-HT_2A_ receptors [126]. Furthermore, the modulatory effects of CBG on CBD as evidenced by Rock and colleagues [121] shows the potential of CBG to modulate other compounds. This could be helpful in both treating seizures directly, and mitigating the side effects of particular AEDs, which currently have deleterious effects to patients across the lifespan [127].

Cascio and colleagues [122] have also observed that at low doses, rather than acting as a 5-HT_2A_ receptor antagonist, CBG stimulates the binding of ligands and receptors. At low micromolar concentrations, CBG was found to stimulate the binding of GTPyS in rodent brain tissue, with this stimulation ceasing at higher concentrations. This low dose stimulation of binding observed with CBG further demonstrates the potential of this compound to be utilised co-operatively with other phytocannabinoids or AEDs to tackle TRE across populations. This molecular diversity of CBG was further delineated by Cascio and colleagues who provided evidence that CBG activates α_2_-adrenoreceptors and blocks activity at 5-HT_1A_ receptors [122]. This activation of α_2_-adrenoreceptors was linked to the possibility that CBG inhibits the neuronal uptake of noradrenaline. Also, the antagonistic activity of CBG observed at the 5-HT_1A_ receptor echoes previous conclusions on CBG activity at serotonin receptors. This specific functionality of these receptors further adds to the anticonvulsant potential of CBG, particularly its ability to modulate other compounds. Socala and colleagues [128] have demonstrated that CBD can enhance various AEDs through such modulation, concluding that more interactional research between other phytocannabinoids and AEDs is warranted.

With CBGs novel molecular targets being delineated, and evidence pointing to its ability to enhance binding across dosage level, it is fruitful to begin investigating CBG as a potential enhancer of other compounds, akin to the established ‘entourage effect’ [129]. Such research has been recently conducted in mood disorders, with cannabinoids enhancing the therapeutic efficacy of terpenes [130].

### 3.5. Overview of Phytocannabinoids

CBD has the most significant amount of evidence available pointing towards its anticonvulsant capability, followed by CBDV. The lack of analyses on Δ^9^-THCV and CBG poses a significant limitation in our ability to draw conclusions on the efficacy of these phytocannabinoids in epilepsy. Researchers must focus on increasing the evidence base of previously under-studied phytocannabinoids. Through use of varied animal models and specific experimental designs the evidence base for under-researched phytocannabinoids can achieve a level of clarity where truly evidence-based conclusions can be drawn. While direct anticonvulsant effects are a significant focus of this review, the neuroprotective effects of these compounds also require attention as they add significantly to the overall profile of phytocannabinoids in relation to neurodegeneration and epilepsy treatment. The next section of this review will detail these neuroprotective effects.

## 4. Neurogenerative/Neuroprotective Potential of Phytocannabinoids

In tandem with potential efficacy to reduce the symptoms of epilepsy, such as seizure, hypotheses have also been put forth that ECS modulators affect neurogenesis and protection. This potential to enable these neurogenerative functions further adds to the profile of phytocannabinoids, making them attractive candidates for epilepsy treatment [131].

Aguado [132] demonstrated that endocannabinoid signalling modulates neural progenitor (NP) cell differentiation *via* the promotion of astroglial differentiation in nascent neural cells. This discovery of NP cells has provided a molecular basis for the process of neurogenesis [133]. Research has demonstrated that plant-derived cannabinoids may modulate deeper neurogenic processes via the ECS [134], with particular interest developing around the role of phytocannabinoids in the process of cell proliferation and survival [135]. This complex physiological regulation of neurogenesis through compounds that directly target the ECS [135] makes phytocannabinoids, such as CBD and CBG, novel targets for treating neurodegeneration.

For example, repeated administration of CBD (30 mg/kg) intraperitoneally over a 14-day timeframe increased hippocampal neurogenesis in rodents [136]. This neurogenic effect of CBD was mediated by CB_1_ receptors, with the administration of CBD causing increases in the levels of the endogenous ECB ligand AEA in the hippocampus. Campos and colleagues [136] demonstrated that CBD administration facilitates proneurogenic action by facilitating endocannabinoid-mediated signalling. This was further confirmed by the same research group [137] where evidence of the neurogenerative effects of CBD in neurological disorders was reviewed. It was concluded that CBD essentially mitigates brain damage associated with neurodegeneration, whilst it simultaneously affects synaptic plasticity and neurogenesis. The mechanisms by which these processes occur are unclear and numerous pathways are postulated as explanatory.

Marchalant and colleagues [138] demonstrated the mediation of the ECS in these processes further. Using the cannabinoid agonist WIN-55212-2 in adult (3 month) male Sprague-Dawley rats at specific doses (0.5 or 0.1 mg/kg/day), the researchers observed that the cannabinoid agonist affected microglia activation. The same research group [139] subsequently replicated these results through the comparison of young-adult (3 month) Sprague-Dawley rats with older-adults of the same species and strain (23 months). In this instance neurogenerative functions were seen across age groups, however, the older sample (23 months) of rats showed significantly less positive effects towards WIN-55212-2 compared to younger samples. WIN-55212-2 restored neurogenesis across samples, but age was shown to be confounding the process. Research [138,139] demonstrated that neurogenesis can be significantly increased by low, continuous doses of a cannabinoid receptor agonist across populations. These results demonstrate the ability of ECS modulators to promote neurodevelopment, with this prevention and active addressing of brain damage being considered antiepileptogenic in cases [140]. Results also point to the salience of such administration in younger samples where it appears to have superior effect on neurogenerative processes.

More recently, a CBG derivative (VCE-003.2) was demonstrated to improve neurogeneration in the striatum of rodents. Researchers showed that VCE-003.2 protected the striatum from damage associated with neurological disorders whilst also attenuating neuroinflammation [141]. This ability of an ECS modulator to promote neurogenerative processes via the modulation of endocannabinoid signalling provides a rationale to investigate the potential role other ECS modulators may play in neurogenerative and protective processes, this being particularly salient in younger adult and adolescent populations [142].

Research has shown that the cannabinoid agonist WIN-555212 at specific doses (0.1 mg/kg) was found to provide robust neuroprotection to 7-day old Wistar rats through the activation of CB_1_ and CB_2_ receptors [142]. Through modulation of the ECS, neuroprotection was observed in these neonate rodents. The ability of agonist activity at CB_1_ and CB_2_ receptors to bring about neuroprotection *in vivo* within a neonate sample provides preliminary evidence of the potential for ECS modulators to bring about these neuroprotective effects in younger human populations. Similarly, these effects were also demonstrated in neonate piglets, with CBD bringing about neuroprotective effects [143]. CBD was observed to be neuroprotective in neonate piglets (3–5 days old) at specific doses (0.1 mg/kg). Interestingly, these effects were also free of any adverse side effects [143]. These observations of the neurogenerative and protective effects of ECS modulators in younger developing brain provide preliminary evidence, which warrants further investigation, specifically with other phytocannabinoid compounds.

## 5. Discussion

Phytocannabinoids have demonstrated therapeutic efficacy across numerous neurological disorders [144]. Their ‘promiscuous’ pharmacology is essentially responsible for their wide therapeutic spectrum [145]. This review has provided collated evidence pointing to the potential of three under-analysed phytocannabinoids (CBDV, THCV, CBG), which could act as effective anticonvulsants. Based on their molecular targets, coupled with specific preclinical conclusions, there is a necessity to investigate these compounds further, with this review promoting further investigation through the lens of epilepsy. This is necessary particularly in younger populations. Except for seizure reduction, it has also been demonstrated that these phytocannabinoids affect neurogenerative and protective processes due to their inherent ability to modulate ECS signalling [137].

Overall, there is not enough evidence to conclusively label the aforementioned phytocannabinoids (except for CBD, which has received extensive attention) as thoroughly effective anticonvulsants in human populations. There is a dearth of research specifically focusing on these compounds in epilepsy. Therefore, more analyses must be conducted on these compounds, specifically in relation to TRE in younger populations. CBD is actively tackling the problem of treatment resistance amongst adolescent and younger adult populations with the approval of Epidiolex^®^. This approval came because of observed efficacy in treating resistant forms of epilepsy, such as LGS and DS across the population [37,38,97]. This therapeutic efficacy, which was observed in CBD came because of robust preclinical and clinical research. Similarly, if other phytocannabinoids, such as CBDV, Δ^9^-THCV and CBG are further studied for their potential as AEDs, more specific research must be conducted on these compounds. Going forward it is about designing robust, population-specific experiments, which will truly determine whether these under-explored phytocannabinoids (CBDV, Δ^9^-THCV, CBG) hold similar therapeutic potential to CBD as anticonvulsants.

Following CBD, CBDV has received the most attention in relation to epilepsy treatment. A phase II trial has demonstrated the pharmacokinetic safety of CBDV in human populations, but CBDVs efficacy as an anticonvulsant was not significant [93]. However, numerous critiques of this clinical trial jeopardise its ability to conclusively state the therapeutic efficacy of CBDV as an anticonvulsant. The primary critique is surrounding the use of a pure CBDV extract. The inefficacy of purified cannabinoids was previously observed in CBD trials, with the use of purified CBD causing significantly less difference in seizure activity compared with CBD-rich formulations [77]. This is not to assume that because a CBD-rich formulation worked, that a CBDV-rich formulation will also provide anticonvulsant characteristics, rather that because it was observed that compound formulation effects compound efficacy, we must rectify the limitations of previous trials on CBDV.

Currently, in relation to CBDV, it can be concluded that not enough research has been conducted in human participants to be truly conclusive in its therapeutic efficacy for patients with epilepsy. There is also a need for further characterisation of CBDVs effects in younger populations where various forms of epilepsy are present. These different epilepsy types can begin in infancy, and persist during a person’s development into adolescence and young adulthood [146]. This paucity of information about CBDVs efficacy in the immature, still developing brain of younger populations further highlights that more specific analyses are warranted. Research has begun recalibrating and addressing these unknowns in younger populations. For example, in the preclinical arena, neonatal rodents have become a sample of interest in order to model CBDVs efficacy in an environment akin to that of human neonates [146]. More specific studies such as this are warranted, with a focus on other developmental populations such as adolescence or young adulthood. Researchers concluded that CBDVs efficacy in infancy may be less promising than other early stages of development such as childhood or adolescence [146]. It is hoped that clinical research on CBDV can begin to focus on these subpopulations as previously called for [146]. With this focus, methodological issues discovered in previous clinical trials that were rectified in the CBD case, can be rectified in the case of CBDV [77].

Regarding Δ^9^-THCV, the literature is more focused on mapping its mechanisms of action *versus* analysing its efficacy as an anticonvulsant. This is understandable due to Δ^9^-THCVs novel status as a drug. However, its efficacy in treating an array of other disorders should be prompting researchers to analyse its efficacy in epilepsy [116,118]. Currently, there is a major dearth of research analysing this compound’s efficacy as an anticonvulsant which must be rectified. Preclinical research has preliminarily demonstrated its efficacy across animal models of epilepsy [94]. This work should be taken up with a more specific focus on replicating these results across varying models of epilepsy, as well as across different developmental samples. Δ^9^-THCV research must ultimately aim for replicating anticonvulsant effects across different models of seizure. Thus far, the scope of Δ^9^-THCVs efficacy is limited by the lack of preclinical analyses across populations and models of epilepsy.

Similarly, the evidence supporting CBG anticonvulsant properties are also sparse. Therefore, concluding presently that the compound acts as an effective anticonvulsant would be tenuous. Preliminary evidence does, however, necessitate further exploration of this compound in relation to epilepsy. CBGs clear ability to modulate the effects of other phytocannabinoids such as CBD [121] also warrants a specific investigation. CBG has potential to act as a compound delivered in tandem with other phytocannabinoids or standard AEDs. A compound such as this, that is capable of managing side effects, is essential across populations. The potential neurogenerative effects of CGB [141] should also be given more attention.

## 6. Conclusions

In conclusion, there are many unknowns in relation to the exact mechanisms by which the above covered phytocannabinoids bring about anticonvulsant effects. However, these unknowns have not stopped CBD from becoming an approved therapy to treat epilepsy. Any conclusions of this review that point to the therapeutic potential of phytocannabinoids in epilepsy are ultimately tentative, and only meant to draw further analytical attention to the potential these compounds hold. Based on this review, it can be concluded that the novel phytocannabinoids deserve further attention in order for the field to be decisive in their conclusions surrounding these compounds and epilepsy treatment. By no means is this review attempting to position phytocannabinoids as the panacea that epilepsy treatment currently needs. Rather, it has attempted to gauge the scientific literature’s current understanding of under-researched compounds, which may provide much needed therapeutic care to minorities of patients suffering with untreatable epilepsy. While further investigation of these phytocannabinoids is key, consideration of their limitations is also pertinent going forward. The lack of psychotropic activity that characterises these compounds should not be an excuse to disregard research on the safety profile of these compounds. With CBD as an exception, there is currently not enough data relating to the safety characteristics of the other phytocannabinoids mentioned in this review. Couple this lack of safety data with the societal stigma surrounding these compounds and it becomes clear that they are not without their limitations from various perspectives including political and law-based fields. These limitations across perspectives must be addressed to guide future use of these compounds in a safe and effective manner. As we are currently in the interim phase of the next advance in AED medication, researchers must do all they can to provide an understanding of these alternate therapeutic means to those mainstream methods which have failed. Toward this end, we should be able to form evidence-based conclusions surrounding the efficacy and safety of various phytocannabinoids in treating epilepsy, thus addressing current gaps and limitations in our collective understanding of these compounds across society. The stark reality faced daily by treatment-resistant individuals with epilepsy is enough to warrant such investigation.

## Figures and Tables

**Table 1:** Data about specific anticonvulsant effects of phytocannabinoids.

Phytocannabinoid	Dose	Species	Effect	Traditional AED in Tandem	Reference
***Cannabidiol (CBD)*** (i) **Animals**	12 & 17 mg/kg	Rats	↑	✓	[81]
	60 mg/kg	Albino mice (m)	↑	✗	[82]
	1, 10 & 100 mg/kg	Wistar rats (m)	↑	✗	[83]
	2 μL (1 μL per side)	NMRI mice (m)	↑	✗	[78]
	10, 20 & 50 mg/kg	Sprague-dawley rats (m)	↑	✗	[84]
(ii) **Humans**	200 mg/kg	Epilepsy Patient Volunteers	↑	✓	[89]
	200–300 mg/kg	Epilepsy Patients (Ages 14–49 years)	↑	✗	[90]
	2–50 mg/kg	Epilepsy Patients (Ages 1–30 years)	↑	✓	[15]
	20 mg/kg	Children and Young adults	↑	✓	[37]
	10 & 20 mg/kg	Epilepsy Patients (Ages 2–55 years)	↑	✓	[38]
***Cannabidivarin (CBDV)*** (i) **Animals**	50–200 mg/kg	DBA/2 mice, CD-1 mice & Wistar rats (m)	↑	✓	[91]
	50–422 mg/kg	Wistar rats, MF1 mice & DBA/2 mice (m)	↑	✗	[92]
(ii) **Humans**	400 mg/kg	Adult Epilepsy Patients	—	✓	[93]
***Delta-9-tetrahydrocannabivarin*** ( **Δ*^9^-THCV)*** (i) **Animals**	10–50 μM & 0.025–2.5 mg/kg	Wistar rats (m)	↑	✗	[94]
***Cannabigerol (CBG)*** (ii) **Animals**	50–200 mg/kg	Wistar rats (m)	—	✗	[95]

(↑): significant effect. (—): no significant effect. (✓): traditional anti-epileptic drug (AED) delivered with phytocannabinoid. (✗): no traditional AED delivered with phytocannabinoid.
